# Age and Surgical Complexity impact on Renoprotection by Remote Ischemic Preconditioning during Adult Cardiac Surgery: A Meta analysis

**DOI:** 10.1038/s41598-017-00308-3

**Published:** 2017-03-16

**Authors:** Chenghui Zhou, Heerajnarain Bulluck, Nengxin Fang, Lihuan Li, Derek J. Hausenloy

**Affiliations:** 1 Department of Anesthesiology, State Key Laboratory of Cardiovascular Disease, Fuwai Hospital, National Center for Cardiovascular Disease, Chinese Academy of Medical Sciences and Peking Union Medical College, Beijing, 100037 China; 2 The Hatter Cardiovascular Institute, University College London, 67 Chenies Mews, London, WC1E 6HX UK; 3 The National Institute of Health Research University College London Hospitals Biomedical Research Centre, London, UK; 4 National Heart Research Institute Singapore, National Heart Centre Singapore, Singapore, Singapore; 5 Cardiovascular and Metabolic Disorders Program, Duke-National University of Singapore, Singapore, Singapore

## Abstract

We aimed to conduct an up-to-date meta-analysis to comprehensively assess the renoprotective effect of remote ischemic preconditioning (RIPC) in patients undergoing adult cardiac surgery. 21 randomized controlled trials (RCTs) with a total of 6302 patients were selected and identified. Compared with controls, RIPC significantly reduced the incidence of acute kidney injury (AKI) [odds ratio (OR) = 0.79; P = 0.02; I^2^ = 38%], and in particular, AKI stage I (OR = 0.65; P = 0.01; I^2^ = 55%). RIPC significantly shortened mechanical ventilation (MV) duration [weighted mean difference (WMD) = −0.79 hours; P = 0.002; I^2^ = 53%), and reduced intensive care unit (ICU) stay (WMD = −0.23 days; P = 0.07; I^2^ = 96%). Univariate meta-regression analyses showed that the major sources of heterogeneity for AKI stage I were age (coefficient = 0.06; P = 0.01; adjusted R2 = 0.86) and proportion of complex surgery (coefficient = 0.02; P = 0.03; adjusted R2 = 0.81). Subsequent multivariate regression and subgroup analyses also confirmed these results. The present meta-analysis suggests that RIPC reduces the incidence of AKI in adults undergoing cardiac surgery and this benefit was more pronounced in younger patients undergoing non-complex cardiac surgery. RIPC may also shorten MV duration and ICU stay. Future RCTs tailored for those most likely to benefit from RIPC warrants further investigation.

## Introduction

Acute kidney injury (AKI) occurs in up to 30%^1^ of patients undergoing adult cardiac surgery, and it is associated with prolonged respiratory support and intensive care unit (ICU) stay, may increase the risk of short-term and long-term death^2–4^, especially in those requiring renal replacement therapy (RRT)^5^. Moreover, with increasing morbidity (such as advanced age, diabetes mellitus, and complex surgical procedures) in this population, postoperative AKI is becoming an important issue in adult patients undergoing cardiac surgery^6, 7^.

Remote ischemic preconditioning (RIPC) is a noninvasive, feasible and low-cost approach elicited by several brief episodes of ischemia and reperfusion (I/R) in a remote organ (a limb using a blood pressure cuff in this study) to offer protection from subsequent ischemic injury^8^. RIPC has proven to be beneficial to protect against I/R injury of various organs^9^ including the kidney^10–13^ in numerous animal studies. In human, RIPC has also been shown to prevent reperfusion-induced endothelial dysfunction^14–16^, and offers a promising strategy for reducing the burden associated with AKI in patients undergoing cardiac surgery.

Several randomized controlled trials (RCTs)^17–22^ have reported on the impact of RIPC on preventing AKI, but the results are mixed. Recently, several striking large-scale RCTs^23–27^ with mixed findings have added to the available evidence for the renoprotective effect of RIPC in adult cardiac surgery. Therefore, we aimed to conduct an up-to-date meta-analysis to comprehensively evaluate the effect of RIPC on the incidence of AKI and identify the related potential influential factors in adults undergoing cardiac surgery.

## Results

### Study characteristics

**Figure 1:**
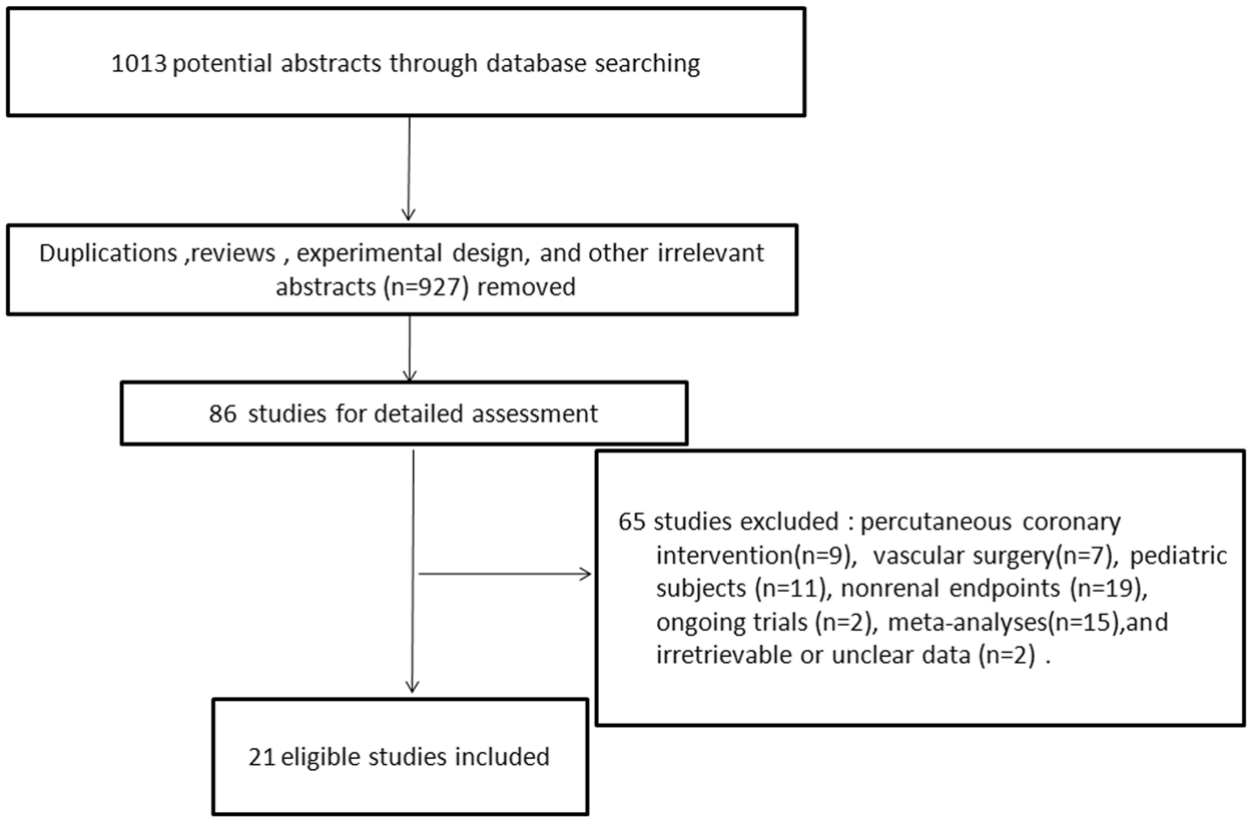
Screening and selection process of eligible RCTs for inclusion in this meta-analysis according to PRISM.

Figure 1 shows the Preferred Reporting Items for Systematic reviews and Meta-Analyses (PRISMA) flow chart for the RCTs screening and selection process for inclusion in this study. 21 RCTs^17–37^ with a total of 6302 patients met the inclusion criteria. 6 RCTs were conducted for isolated coronary artery bypass graft (CABG)^17, 22, 28–31^, 5 RCTs were done in isolated valve surgery^20, 21, 34, 35, 37^, and 10 RCTs included a combination of CABG and valve surgery^18, 19, 23–27, 32, 33, 36^. The ischemic protocol (cycles × I/R) was 3 × 5 min/5 min in 11 RCTs^17–19, 22, 26, 28, 29, 32, 35–37^, 4 × 5 min/5 min in 7 RCTs^24, 25, 27, 30, 31, 33, 34^, 3 × 10 min/10 min in 2 RCTs^20, 21^, and 2 × 5 min/5 min in 1 RCT^23^. The upper limb was used in 11 RCTs^17, 19, 22–29, 32, 35^, the lower limb in 3 RCTs^18, 20, 37^, the thigh in 6 RCTs^21, 30, 31, 33, 34, 36^, the combined of upper limb and thigh in 1 RCT^23^. The incidence of AKI was reported in 17 RCTs^17–27, 31–36^ (AKI stage I in 11 RCTs^17–19, 22–27, 32, 36^), the need for RRT in 19 RCTs^17–20, 22–33, 35–37^, mortality in 19 RCTs^17–19, 21–36^, MV duration in 13 RCTs^17, 20–22, 26–29, 31–35^, ICU stay in 16 RCTs^17, 20–23, 25–29, 31, 33–37^, and hospital length of stay (LOS) in 16 RCTs^17–23, 25–28, 31, 33, 34, 36, 37^. 19 RCTs^17–21, 23–28, 30–37^ had a Jadad score of more than 3. Further details of RCTs characteristics and the RIPC protocol used in each RCT are provided in Tables 1 and 2.

**Table 1:** Study design in all included RCTs.

Study	Country	Surgery	Pts. No. RIPC vs Ctrl	RIC protocol		RIC initiation to CPB	Placebo Control	Renal Endpoints	Baseline Creatinine level (mg/dl)	AKI Definition	F-up	Jadad score
				Cycles × I/R	Cuff pressure							
Rahman^17^	UK	CABG (On)	42 vs 38	3 × 5 min/5 min at upper limb	200 mmHg	74 mins	Yes	AKI, RRT, Mortality	1.10	SCr↑ >0.5 mg/dl	30 d	5
Thielmann^28^	German	CABG (On)	27 vs 26	3 × 5 min/5 min at upper limb								
Venugopal^19^	UK	Combined	38 vs 40	3 × 5 min/5 min at upper limb	200 mmHg	<45~60 mins	Yes	AKI, RRT, Mortality	0.95	AKIN	30 d	4
Zimmerman^18^	USA	Combined	59 vs 59	3 × 5 min/5 min at thigh	200 mmHg	N.A	No	AKI, RRT, Mortality	0.94	AKIN	In-hospital	5
Choi^20^	Korea	Valve	38 vs 38	3 × 10 min/10 min at thigh	250 mmHg	>70 mins	Yes	AKI, RRT	0.915	AKIN	In-hospital	5
Lomivorotov^29^	Russian	CABG (On)	40 vs 40	3 × 5 min/5 min at upper limb	200 mmHg	30~50 mins	Yes	RRT	N.A	RRT	In-hospital	1
Lucchinetti^30^	Canada	CABG (On)	27 vs 28	4 × 5 min/5 min at thigh	300 mmHg	N.A	Yes	RRT	1.01	RRT	6 mon	5
Hong^31^	Korea	CABG (Off)	35 va 35	4 × 5 min/5 min at thigh	200 mmHg	18 mins	Yes	RRT, Mortality	1.10	RRT	30 d	3
Kim^21^	Korea	Valve	27 vs 27	3 × 10 min/10 min at thigh	250 mmHg	Pre- plus Post-CPB	Yes	AKI, RRT, Mortality	N.A	AKIN	In-hospital	5
Young^32^	New Zealand	Combined	48 vs 48	3 × 5 min/5 min at upper limb	200 mmHg	N.A	Yes	AKI, RRT, Mortality	1.10	RIFLE	30 d	5
Gallagher^22^	UK	CABG	43 vs 43	3 × 5 min/5 min at upper limb	50 mmHg > SBP	N.A	Yes	AKI, RRT, Mortality	1.37	AKIN	30 d	2
Candilio^23^	UK	Combined	57 vs 54	2 × 5 min/5 min at upper limb and thigh	200 mmHg	<45 mins	Yes	AKI, RRT	N.A	AKIN	In-hospital	5
Hong^33^	Korea	Combined	644 vs 636	4 × 5 min/5 min at thigh	200 mmHg	N.A	Yes	AKI	N.A	AKIN	In-hospital	5
Hu^34^	China	Valve	101 vs 100	4 × 5 min/5 min at thigh	600 mmHg	Post-CPB	Yes	AKI, Mortality	0.83	AKIN	In-hospital	4
Pinaud^35^	France	Valve	50 vs 49	3 × 5 min/5 min at upper limb	200 mmHg	91 mins	Yes	AKI, RRT	N.A	AKIN	In-hospital	3
Hausenloy^25^	UK	Combined	749 vs 772	4 × 5 min/5 min at upper limb	200 mmHg	105 min	Yes	AKI, RRT, Mortality	N.A	KDIGO	In-hospital	5
Zarbock^26^	German	Combined	120 vs 120	3 × 5 min/5 min at upper limb	200 mmHg or 50 mmHg > SBP	N.A	Yes	AKI, RRT, Mortality	1.15	KDIGO	In-hospital	5
Meybohm^24^	German	Combined	692 vs 693	4 × 5 min/5 min at upper limb	≥200 mmHg or 15 mmHg > SBP	N.A	Yes	RRT, Mortality	N.A	RIFLE	In-hospital	5
Cao^37^	China	Valve	30 vs 30	3 × 5 min/5 min at lower limb	200 mmHg	N.A	Yes	RRT	N.A	RRT	In-hospital	3
Walsh^36^	Canada/US/India/China	Combined	128 vs 130	3 × 5 min/5 min at thing	300 mmHg	N.A	Yes	AKI, RRT, Mortality	1.07	AKIN	6 mon	5
Kim^27^	Korea	Combined	80 vs 80	4 × 5 min/5 min at upper limb	200 mmHg	29.4 h	Yes	AKI, RRT, Mortality	0.9	AKIN	In-hospital	5

Note: RCT, randomized controlled trials; CABG, coronary artery bypass graft; I/R, ischemia/reperfusion; SBP, systolic blood pressure; atm, atmosphere; AKI, acute kidney injury; RRT, renal replacement treatment; SCr, serum creatinine; eGFR, estimated glomerular filtration rate; N.A, not available; AKIN, Acute Kidney Injury Network; RIFLE, Risk, Injury, Failure, Loss of renal function and End-stage renal disease; KDIGO, Kidney Disease: Improving Global Outcomes; RIPC, remote ischemic preconditioning; Ctrl, control.

**Table 2:** Patient characteristics in all included randomized trials.

Substudy	Age	Male (%)	Pre-MI (%)	DM (%)	HT (%)	Dyslipidemia (%)	Renal dysfunction (%)	CPB duration (min)	Baseline LVEF (%)	Complex Surgery (%)	CABG (%)	Volatile Anesthetic (%)	Aspirin (%)	ACEI (%)	β-blockers (%)	Statins (%)
Rahman^17^	64.0	88.5	0.0	0.0	59.3	74.1	N.A	98.0	60.1	0.0	100.0	98.1	88.3	64.8	80.9	90.7
Thielmann^28^	63.7	85.0	37.7	0.0	92.5	84.9	N.A	109.5	1.5(<35%)	0.0	100.0	100.0	83.0	64.2	75.5	64.2
Venugopal^19^	65.0	82.0	23.0	0.0	65.4	75.6	N.A	85.4	1.0(<35%)	14.1	96.0	61.5	66.7	65.4	55.0	79.5
Zimmerman^18^	63.5	68.6	N.A	22.5	47.0	N.A	16.1(eGFR<60)	114	10.2(<35%)	11.0	40.0	100.0	N.A	14.0	N.A	N.A
Choi^20^	58.5	39.5	23.5	7.0	9.0	N.A	11.0(eGFR<60)	138.5	61.5	23.5	0.0	100.0	N.A	44.7	20.0	7.9
Lomivorotov^29^	57.3	96.1	N.A	0.0	N.A	N.A	N.A	64.5	59.0	0.0	100.0	100.0	N.A	56.6	86.8	N.A
Lucchinetti^30^	60.5	91.0	41.8	0.0	70.9	85.5	N.A	101.0	52.0	0.0	100.0	100.0	N.A	51.0	91.0	96.4
Hong^31^	64.7	72.9	N.A	35.7	68.6	17.1	0.0	54.0	0.0(<30.0%)	0.0	100.0	0.0	94.3	54.3	64.3	72.9
Kim^21^	57.5	55.6	N.A	13.0	33.3	N.A	0.0	127.5	64.5	48.1	0.0	N.A	N.A	11.1	22.2	5.6
Young^32^	66.4	62.5	27.8	N.A	N.A	60.4	N.A	111.1	2.0(<30.0%)	31.3	57.3	100.0	N.A	52.1	66.7	60.4
Gallagher^22^	70.8	80.2	52.3	64.0	82.6	77.9	N.A	94.0	52.0/10.5(<35%)	5.8	96.5	87.2	96.5	79.1	35.0	N.A
Candilio^23^	65.5	78.1	28.7	29.2	78.8	74.2	0.0	93.2	4.5(<30%)	11.8	62.4	96.1	77.5	66.3	62.9	80.9
Hong^33^	60.8	61.3	7.3	30.2	48.6	53.8	3.1	159.7	57.0	19.7	50.8	N.A	48.3	39.1	42.7	N.A
Hu^34^	47.1	37.8	0.0	0.0	0.0	N.A	0.0	81.3	0.0(<35%)	39.3	0.0	100.0	N.A	N.A	N.A	N.A
Pinaud^35^	74.4	51.5	0.0	14.1	77.8	53.5	N.A	81.4	65.6	0.0	0.0	100.0	18.2	20.2	28.3	40.4
Hausenloy^25^	76.2	70.8	39.5	25.7	74.5	69.8	0.0	70.0	11.6(<35%)	50.2	N.A	40.2	78.4	60.3	64.0	79.7
Zarbock^26^	70.4	62.9	0.0	37.5	96.7	N.A	30.9	118.0	15.0(<35%)	46.3	N.A	100.0	59.6	60.0	60.8	68.8
Meybohm^24^	66.0	74.2	28.9	24.8	N.A	N.A	11.2	115.0	0.0(<35%)	27.2	N.A	2.7	N.A	52.7	63.2	65.5
Cao^37^	53.0	48.3	N.A	0.0	N.A	N.A	N.A	115.0	51.0	N.A	N.A	N.A	0.0	N.A	N.A	0.0
Walsh^36^	72.2	58.5	29.4	30.6	N.A	N.A	3.9	137.6	N.A	32.2	57.0	83.7	N.A	N.A	N.A	N.A
Kim^27^	62.3	53.1	N.A	0.0	34.4	N.A	0.0	230.9	58.5	36.3	6.3	0.0	N.A	N.A	N.A	N.A

Note: Pre-MI, previous myocardial infarction; DM, diabetes mellitus; HT, hypertension; CPB, cardiopulmonary bypass; eGFR, estimated glomerular filtration rate; LVEF, left ventricular ejection fraction; CABG, coronary artery bypass graft; ACEI, angiotensin-converting enzyme inhibitor; N.A, not available.

### Effect of RIPC on the incidence of AKI, RRT, and Mortality

**Figure 2:**
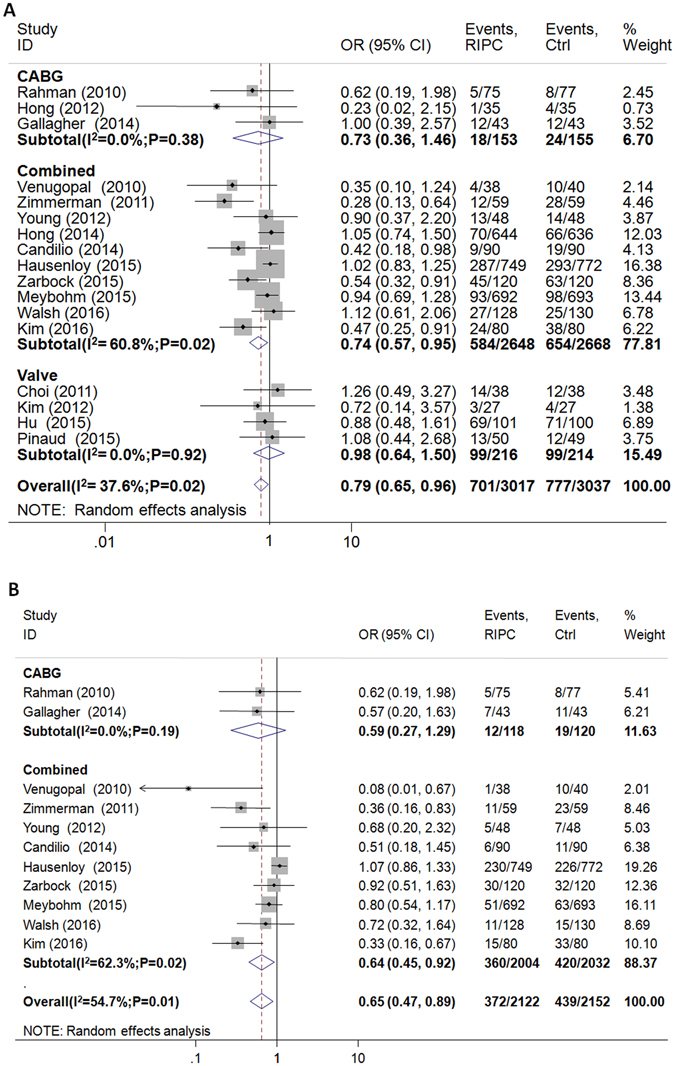
Forest plot of the effect on RIPC on (**A**) AKI and (**B**) AKI stage I.

AKI was reported in 6054 study subjects, and the overall incidence was 25% (707/3017 in RIPC group, 777/3037 in control group). Postoperative incidence of AKI was significantly reduced by RIPC (17 RCTs; odds ratio (OR) = 0.79; 95% CI, 0.65 to 0.96; P = 0.02; I^2^ = 38%; Fig. 2A). There was no evidence of publication bias (Begg’s test P = 0.22; Egger’s test P = 0.32).

For AKI, stage I the overall incidence was 19% (372/2122 in RIPC group, 439/2152 in control group). RIPC significantly reduced the risk of AKI stage I (11 RCTs; OR = 0.65; 95% CI, 0.47 to 0.89; P = 0.007; I^2^ = 55%; Fig. 2B) with no significant publication bias (Begg’s test P = 0.19; Egger’s test P = 0.08). There was no difference in the incidence of AKI stage II or stage III between the 2 groups as shown in Table 3.

**Table 3:** Pooled analysis of the postoperative primary and second endpoints.

Endpoints	References	RIPC	Control	Pts with complete data	OR (95% CI)	WMD (95% CI)	P value
AKI	17–27, 31–36	707/3017(23.4%)	777/3037(25.6%)	96.06%	0.79(0.65, 0.96)	/	0.02
AKI stage I	17–19, 22–27, 32, 36	372/2122(17.5%)	439/2152(20.4%)	67.79%	0.65(0.47, 0.89)	/	0.007
AKI stage II	18, 19, 22–27, 32, 36	105/2046(5.1%)	100/2074(4.8%)	65.38%	1.07(0.81,1.42)	/	0.64
AKI stage III	17–20, 22–33, 35–37	89/3013(3.0%)	94/3034(3.1%)	95.95%	0.92(0.58,1.45)	/	0.71
RRT	17–20, 22–33, 35–37	89/3013(3.0%)	94/3034(3.1%)	95.95%	0.92(0.58,1.45)	/	0.71
Mortality (30-day)	17–19, 21, 22, 24, 26–29, 31–35	31/2079(1.5%)	32/2073(1.5%)	65.89%	0.96(0.58, 1.61)	/	0.89
Mortality (<1 year)	17, 23, 25, 30, 36	60/1069(5.6%)	48/1097(4.4%)	34.37%	1.19(0.62, 2.29)	/	0.60
MV duration	17, 20–22, 26–29, 31–35	1330	1317	42.00%	/	−0.77(−1.32, −0.23)	0.005
ICU stay	17, 20–23, 25–29, 31, 33–37	2277	2293	72.52%	/	−0.23(−0.49, 0.02)	0.07
Hospital LOS	17–23, 25–28, 31, 33, 34, 36, 37	2284	2303	72.79%	/	−0.01(−0.28, 0.25)	0.92

Notes: AKI, acute kidney injury; RRT, renal replacement treatment; MV duration, mechanic ventilation duration; ICU stay, intensive care unit stay; Hospital LOS, hospital length of stay; Pts, patients; OR, odds ratio.

WMD, weighted mean difference; CI, confidence interval; RIPC, remote ischemic preconditioning.

The RRT was reported in 6047 study subjects, and the overall incidence was 3% (89/3013 in RIPC group, 94/3034 in control group). The risk of postoperative RRT was not lowered in the RIPC group (19 RCTs; OR = 0.92; 95% CI, 0.58 to 1.45; P = 0.71; I^2^ = 37%; Table 3).

The 30-day and 1-year mortality data were available in 4152 and 2166 patients and the mortality rates were 1.5% and 5% respectively. There was no significant difference between the RIPC group and the control group for both these endpoints as shown in Table 3.

### Effect of RIPC on MV duration, ICU stay, and hospital LOS

**Figure 3:**
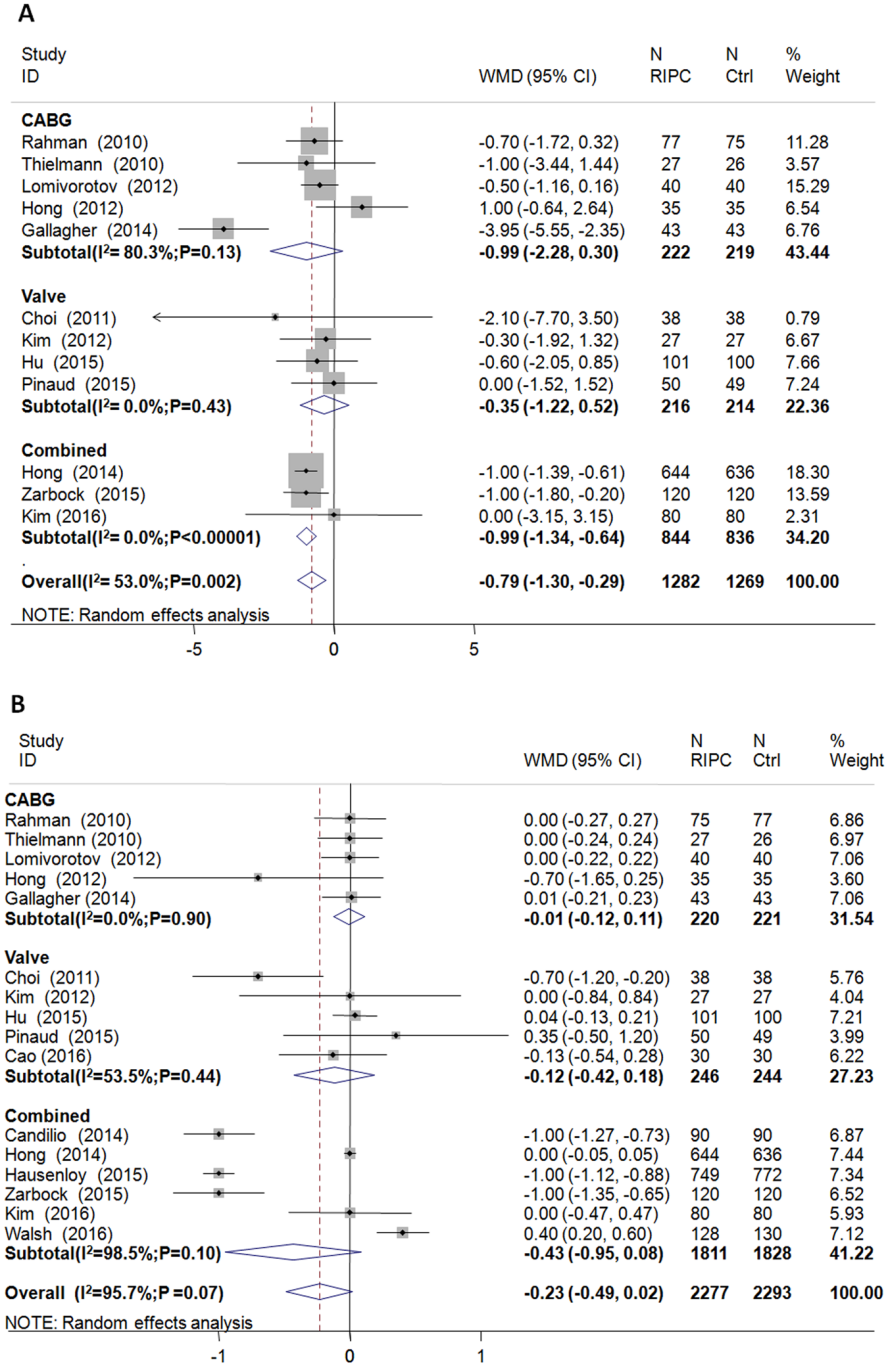
Forest plot of the effect on RIPC on (**A**) MV duration and (**B**) hospital length of stay.

RIPC significantly shortened MV duration by 0.77 hours (13 RCTs; 95% CI, −1.32 to −0.23 hours; P = 0.005; I^2^ = 57%), and there was a trend towards reduced ICU stay by 0.23 days (16 RCTs; 95% CI, −0.49 to 0.02 days; P = 0.07; I^2^ = 96%) (Fig. 3). However, RIPC did not affect hospital LOS (16 RCTs; −0.01 days, 95% CI, −0.28 to 0.25 days; P = 0.92; I^2^ = 45%; Table 4).

**Table 4:** Meta-regression and Subgroup analyses for the potential sources of heterogeneity.

Variables	Endpoint	No. RCTs	Covariate	Coeff./OR/WMD	95% CI	P Value		
***Univariate***				***Coeff.***				***Adjusted R*** ^***2***^
Age (years)	AKI stage I	11	/	0.06	0.02~0.11	0.01		0.86
HT (%)	AKI stage I	8	/	0.02	−0.004~0.04	0.09		0.49
Additive ischemia (%)	AKI stage I	11	/	0.04	0.007~0.07	0.02		0.93
Complex surgery (%)	AKI stage I	11	/	0.02	0.002~0.03	0.03		0.81
***Multivariate***				***Coeff.***				***Adjusted R*** ^***2***^
Age	AKI stage I	9	Previous MI (%)	0.05	−0.01~0.10	0.09		1.00
	AKI stage I	10	Diabetes (%)	0.06	0.005~0.12	0.04		0.81
	AKI stage I	8	HT (%)	0.07	0.01~0.13	0.03		1.00
Complex surgery (%)	AKI stage I	9	Previous MI (%)	0.02	−0.001~0.03	0.06		1.00
	AKI stage I	10	Diabetes (%)	0.02	0.003~0.033	0.03		0.95
***Subgroup***							***I*** ^***2***^	***P***_***Difference***_ ***Value***
				***OR***				
1. Age (years)	AKI stage I	11	/	0.65	0.47~−0.89	0.007	54.70%	<0.00001
≥66.0		6	/	0.95	0.80~1.13	0.58	0.00%	
<66.0		5	/	0.37	0.24~0.58	<0.00001	0.00%	
2. Complex surgery (%)	AKI stage I	11	/	0.65	0.47~−0.89	0.007	54.70%	0.005
≥25%		5	/	0.78	0.57~1.08	0.13	54.00%	
<25%		6	/	0.43	0.27~0.71	0.001	0.00%	

Note: AKI, acute kidney injury; ICU stay, intensive care unit stay; HT, hypertension; previous MI, previous myocardial infarction; LVEF, left ventricular ejection fraction; Coeff., coefficient; WMD, weighted mean difference; CI, Confidence Interval.

### Meta-regression and Subgroup analyses for Potential Sources of Heterogeneity

**Figure 4:**
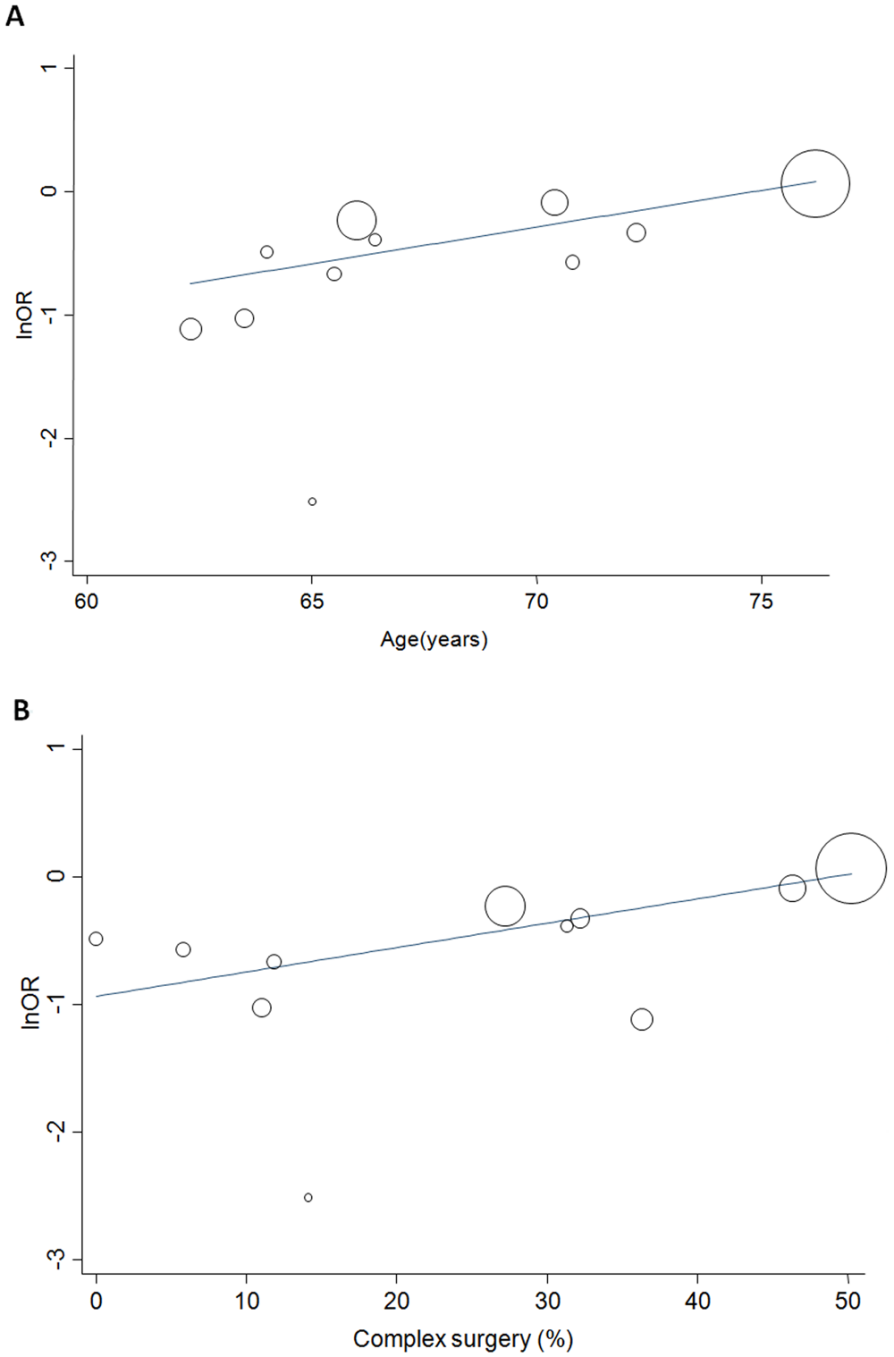
Meta-regression plots on the incidence of AKI stage I against (**A**) age and (**B**) proportion of complex surgery.

Age, male, previous myocardial infarction (MI), diabetes, hypertension, dyslipidemia, renal dysfunction, cardiopulmonary bypass duration, baseline left ventricular ejection fraction, complex surgery, CABG, use of volatile anesthesia, aspirin, angiotensin-converting enzyme inhibitors, beta-blockers, and statins, cumulative duration of preconditioned ischemia, and additive ischemia were included in the random-effect univariate meta-regression analyses for AKI stage I. The major sources of heterogeneity were age (coefficient = 0.06; P = 0.01; adjusted R^2^ = 0.86), hypertension (coefficient = 0.02; P = 0.09; adjusted R^2^ = 0.49), additive ischemia (coefficient = 0.04; P = 0.02; adjusted R^2^ = 0.93), and complex surgery (coefficient = 0.02; P = 0.03; adjusted R^2^ = 0.81) as shown in Table 4. Subsequent multivariate analyses showed that age (coefficient = 0.06; P = 0.01) and complex surgery (coefficient = 0.02; P = 0.03) remained significantly associated with AKI stage I, as shown in the meta-regression plots in Fig. 4 and Table 4. There was a relative reduction in the estimated effect size by 0.06 (natural transformation of OR) per 1-year increase in age and by 0.20 (natural log transformation of OR) per 10% increase in the proportion of complex surgery for AKI stage I by RIPC.

Subgroup analyses showed that RCTs with a mean age of <66 years old had less risk of AKI stage I than those with a mean age of ≥66 years old [OR: 0.37 versus 0.95, P < 0.001 for subgroup difference; Table 4]. Furthermore, RCTs with the proportion of complex surgery being <25% had significantly less AKI stage I than those with the proportion of complex surgery being ≥25% [OR: 0.43 versus 0.78; P = 0.005 for subgroup difference; Table 4].

## Discussion

In this meta-analysis of 21 RCTs involving 6302 adult patients undergoing cardiac surgery, we found that RIPC reduced the incidence of AKI. In addition, RIPC also shortened MV duration and there was a trend towards shorter ICU stay, but heterogeneity among the included RCTs was substantial for the latter. RIPC was more effective at reducing AKI stage I in RCTs with younger patients (<66 years old) and in those RCTs with less complex cardiac surgery (<25%). However, RIPC did not affect AKI stage II and III/requirement for RRT, hospital length of stay, and mortality.

Post-operative AKI in adult cardiac surgery is a common complication, occurring in up to a third of surgical cases^4, 38, 39^. Even minor increase in postoperative serum creatinine level following cardiac surgery has been shown to be associated with increased MV duration^40^, prolonged ICU stay^41^, and the risk of short-term mortality^2, 41, 42^. Although AKI can occur due to numerous reasons and the underlying mechanisms remain unclear, acute tubular necrosis has been implicated as being the predominant pathology^43^. There is currently no effective renoprotective strategy to reduce the burden of AKI in this setting^44^. Several RCTs have investigated the renoprotective effect of RIPC in patients undergoing cardiac surgery but with conflicting results. To minimize heterogeneity due to trial design and patient selection, our study only included RCTs involving adult cardiac surgery, but not in combination with major vascular surgery^45, 46^, pediatric cardiac surgery^46, 47^, percutaneous coronary intervention^47, 48^, or organ transplantation^47^.

We found that RIPC reduced the incidence of AKI stage I and MV duration, and there was a trend towards shorter ICU stay. Our findings are consistent with the RCT by Zarbock *et al*.^26^, which was specifically designed and powered to look at the effect of RIPC on AKI as the primary endpoint in 240 patients. Of note, they only included patients at high risk of AKI. Furthermore, they used volatile anesthesia instead of propofol, the latter of which may potentially attenuate the effect of RIPC^49, 50^. They showed a 15% and 10% absolute risk reduction in the incidence of AKI and the need for RRT, respectively. RIPC also reduced the duration of stay in ICU but there was no difference in overall hospital length of stay^26^.

The risk of death is proportional to the severity of AKI, with the highest rate occurring in patients requiring RRT following adult cardiac surgery^4, 51, 52^. In our analysis, the incidence of RRT was 3.1% and the 30-day mortality was only 1.5% (4152 patients), many of whom presented with normal preoperative serum creatinine level. RIPC did not affect the need for RRT or mortality in our analysis. Thielmann *et al*.^53^ randomized 329 patients undergoing CABG and obtained similar findings to our study for 30-day mortality. However, they found that RIPC reduced 1-year mortality and the result remained significant after 4-year follow-up. Therefore, longer follow-up duration should be considered in future RCTs to see a benefit in mortality.

Translating renoprotective strategies that have shown promise in young and healthy animals into the clinical population with various co-morbidities and/or confounders (such as age^54, 55^, surgical complexity^56^, and previous MI^57^) has proven to be challenging. Our meta-regression analysis showed that age was negatively correlated with the reduction in AKI stage I by RIPC. Likewise, the proportion of complex surgery was negatively correlated with the reduction in AKI stage I. Based on the findings from our study and that of Zarbock *et al*.^26^, whether pre-selecting a younger cohort of patients who are at risk of AKI, undergoing non-complex surgery using volatile anesthesia may more likely show a significant reduction in all stages of AKI by RIPC and eventually improve clinical outcomes, remain to be assessed in future, adequately powered RCTs.

There are several limitations in our study. Firstly, we were unable to access the patient-level data. Therefore, the potential influences of co-morbidities (diabetes^19^, baseline left ventricular ejection fraction^57^, and interval between coronary angiography and surgery^58^) and cardiovascular medications (such as volatile anesthetics^59^ and statins^60^) may have been underestimated. Secondly, AKI was based on different definitions such as AKI Network classification (AKIN), Risk/Injury/Failure/Loss/End-stage (RIFLE) criteria or the Kidney Disease: Improving global Outcomes (KDIGO) classification^61, 62^, and the patient selection, type of surgery and RIPC protocol used were different and may have contributed to the heterogeneity. Thirdly, although we included several recently published large RCTs, the sample size was still relatively small to be adequately powered for hard clinical outcomes. Last but not least, only 11 RCTs qualified for the meta-regression analysis and therefore the conclusions may not be robust but hypothesis generating.

In conclusion, the available evidence from the present meta-analysis indicates that RIPC reduces the incidence of AKI in adults undergoing cardiac surgery and this benefit was more pronounced in younger patients undergoing non-complex cardiac surgery. RIPC may also shorten MV duration, and length of stay in ICU, and this warrants further investigation in future RCTs tailored for those most likely to benefit.

## Methods

### Search strategy and study criteria

This meta-analysis was performed according to the PRISMA statement^63^ as shown in the flow chart in Fig. 1. We did a systematic search in PubMed, EMBase, and Cochrane Library (up to November 2016) using keywords “remote ischemic preconditioning”, “remote ischaemic preconditioning”, “ischemic preconditioning”, “cardiac surgery”, “heart surgery”, “kidney”, and “renal”. Furthermore, editorials and references from included RCTs were manually searched. RCTs published in English and involving adult patients were included. Exclusion criteria were: (1) pediatric cardiac surgery; (2) studies not reporting acute kidney injury (AKI) and RRT during hospitalization.

### Literature review and data extraction

The literature review and data extraction were independently completed by two investigators (J.G. and Y.Z.). Any disagreements were resolved by consensus. Quality assessment was performed according to Jadad score: randomization; blinding; withdrawals and dropouts (a possible score between 0 and 5). Trials with a score of more than 3 were considered as being of high-quality^64^. Data extraction included patient’s age, male gender, history of MI, diabetes, hypertension, dyslipidemia, renal dysfunction, CPB duration, baseline LVEF, type of surgery (complex surgery defined as a combination of valve, CABG, or major vascular surgery), usage of volatile anesthesia, aspirin, angiotensin converting enzyme inhibitors, beta-blockers and statins. Cumulative duration of preconditioned ischemia was calculated multiplying the number of cycles by the ischemic duration (for example, 3*5 min = 15 min for preconditioning with 3 × 5 min ischemia/5 min reperfusion). Additive ischemia^65^ was calculated using cumulative duration of preconditioned ischemia relative to the CPB duration.

### Postoperative Outcomes

The primary endpoints were incidence of AKI as a whole and AKI stage I–III individually, and the definition used by each RCT (AKIN, RIFLE, or KDIGO criteria^61^) was used for this study.

The secondary endpoint included RRT (defined as dialysis or hemofiltration), mechanic ventilation (MV) duration, intensive care unit (ICU) stay, and hospital length of stay (LOS).

### Statistical analysis

For dichotomous outcomes (reported as incidence), we calculated OR with 95% CI. For continuous outcomes (MV duration, duration of stay in ICU and hospital length of stay) reported as mean and standard deviation, the WMD for the pooled estimates with 95% CI were calculated. For RCTs reporting median and interquartile range, or median and range, the method described by Hozo *et al*.^66^ was used to convert to mean and standard deviation. Random-effect model was used in view of differences in patient selection and the RIPC protocol used among the RCTs. Publication bias was assessed by Begg’s test and Egger’s test. Heterogeneity among RCTs was quantified using I^2^ statistics with I^2^ of 0–40%, 30–60%, 50–90% and 75–100% considered as low, moderate, substantial and considerable heterogeneity, respectively, as defined by the Cochrane handbook of systematic reviews^67^ and moderate heterogeneity was considered acceptable. Meta-regression (P < 0.1) and subgroup analysis were conducted for positive results to explore the potential sources of heterogeneity^68^. To reduce the possibility of over-fitting in the multivariate regression model, at least four studies or sub-studies were set for the identification of each influential factor^69, 70^. P < 0.05 (2-sided) was considered to be statistically significant. All statistical analyses were performed in Stata (version 9.0; Stata Corporation, College Station, TX).
